# On the characterization of ultra-precise X-ray optical components: advances and challenges in *ex situ* metrology

**DOI:** 10.1107/S1600577514016221

**Published:** 2014-08-27

**Authors:** F. Siewert, J. Buchheim, T. Zeschke, M. Störmer, G. Falkenberg, R. Sankari

**Affiliations:** a Institut für Nanometer Optik und Technologie, Helmholtz Zentrum Berlin, Albert-Einstein-Strasse 15, Berlin, Germany; b Centre for Material Research and Coastal Research, Helmholtz-Zentrum Geesthacht, Max-Planck-Strasse 1, Geesthacht 21501, Germany; c Photon Science, Deutsches Elektronen-Synchrotron DESY, Notkestrasse 85, Hamburg 22607, Germany; d MAX IV Laboratory, Lund University, Lund SE-22100, Sweden

**Keywords:** synchrotron optics, X-ray optics, metrology for synchrotron optics, slope measurement, NOM, multilayer, focusing mirrors

## Abstract

To fully exploit the ultimate source properties of the next-generation light sources, such as free-electron lasers (FELs) and diffraction-limited storage rings (DLSRs), the quality requirements for gratings and reflective synchrotron optics, especially mirrors, have significantly increased. These coherence-preserving optical components for high-brightness sources will feature nanoscopic shape accuracies over macroscopic length scales up to 1000 mm. To enable high efficiency in terms of photon flux, such optics will be coated with application-tailored single or multilayer coatings. Advanced thin-film fabrication of today enables the synthesis of layers on the sub-nanometre precision level over a deposition length of up to 1500 mm. Specifically dedicated metrology instrumentation of comparable accuracy has been developed to characterize such optical elements. Second-generation slope-measuring profilers like the nanometre optical component measuring machine (NOM) at the BESSY-II Optics laboratory allow the inspection of up to 1500 mm-long reflective optical components with an accuracy better than 50 nrad r.m.s. Besides measuring the shape on top of the coated mirror, it is of particular interest to characterize the internal material properties of the mirror coating, which is the domain of X-rays. Layer thickness, density and interface roughness of single and multilayer coatings are investigated by means of X-ray reflectometry. In this publication recent achievements in the field of slope measuring metrology are shown and the characterization of different types of mirror coating demonstrated. Furthermore, upcoming challenges to the inspection of ultra-precise optical components designed to be used in future FEL and DLSR beamlines are discussed.

## Abstract 2

State-of-the-art *ex situ* metrology for characterizing the quality of ultraprecise reflective synchrotron optics is reported. Beside slope measuring deflecometry the current state of mirror coating technology for single layer and multilayer coatings for very long mirror substrates is discussed.

## 1. Introduction   

**Figure 1:**
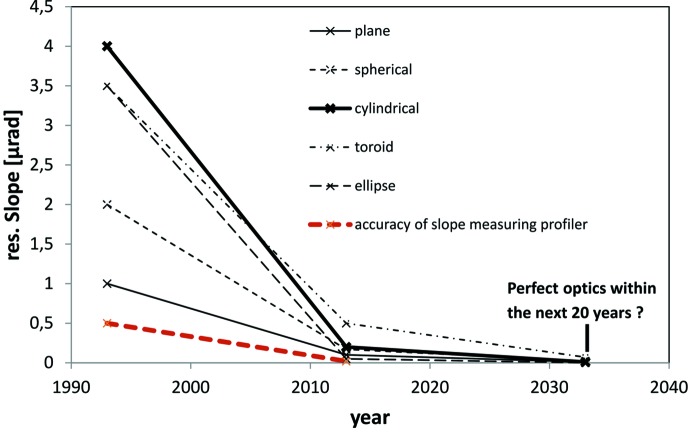
Improvement of mirror quality in terms of slope error during the last two decades (based on data and measurements performed at the BESSY-II Optical Metrology Laboratory).

With the advent of highly brilliant synchrotron sources like the PETRA III storage ring, X-ray free-electron lasers (XFELs) as well as diffraction-limited storage rings (DLSRs) like the new MAX IV laboratory, the requirements for synchrotron optics in terms of precision have significantly increased. Compared with the situation two decades ago, when the first third-generation storage rings came into operation, an improvement of the mirror shape by one order of magnitude in terms of residual slope error has been achieved to date (see Fig. 1 ▶). It is assumed that upcoming developments will improve the quality of optical components within the near future even further. For planar optics, grating blanks of 100 nrad r.m.s. residual slope error, 150 mm in length and a curvature radius larger than 200 km are state of the art (Siewert, 2013 ▶). Future applications require significantly longer grating blanks of excellent quality. Future plane gratings at the European XFEL will have a length of 530 mm and require a radius of curvature of >300 km and 50 nrad r.m.s. for the residual slope deviation (Vannoni *et al.*, 2013 ▶). It is also being discussed to install plane mirrors with of length 800 mm, 50 nrad r.m.s. slope error, 2 nm peak-to-valley (p.v.) figure error and a curvature radius larger than 1000 km in beamlines at the European XFEL (Samoylova *et al.*, 2009 ▶). For non-planar optics the trend is the same: cylindrical gratings designed for the resonant inelastic X-ray scattering (RIXS) experiments at MAX IV require an aperture length of 280 mm with 100 nrad r.m.s. slope error. In addition, elliptical-cylinder-shaped focusing optics of 20 nrad r.m.s. residual slope deviation up to a length of 1000 mm are proposed to focus photons at the Single Particles, Clusters and Biomolecules (SPB) experimental station at the European XFEL (Mancuso *et al.*, 2013 ▶). In contrast to such long focusing mirrors, the length of Kirkpatrick–Baez (KB) mirror substrates at sources like PETRA III or MAX IV will be in the range 100–200 mm (Kalbfleisch *et al.*, 2010 ▶; Johansson, 2014 ▶). Such optical components of elliptical and hyperbolic shape are already available today (Matsuyama *et al.*, 2012 ▶; Siewert *et al.*, 2012 ▶) and allow diffraction-limited focusing of hard X-ray photons within nanometre focus size (Mimura *et al.*, 2010 ▶). All these optical components used in tangential focusing have fairly large radii of curvature. On the other hand, sagittal focusing, which was proven to preserve brilliance very well, especially in the soft X-ray regime, results in highly curved surfaces of cylindrical, toroidal or ellipsoidal form setting new challenges for manufacturing.

Dedicated metrology instrumentation of comparable accuracy has been developed to characterize such optical elements. Second-generation slope-measuring profilers like the Nano­meter Optical component measuring Machine (NOM) (Siewert *et al.*, 2004 ▶; Yashchuk *et al.*, 2010 ▶; Nicolas & Martinez, 2013 ▶; Assoufid *et al.*, 2013 ▶) allow the inspection of reflective optics up to a length of 1.5 m (Alcock *et al.*, 2010 ▶) with an accuracy better than 50 nrad r.m.s. It has taken the place of the well known Long Trace Profiler-II (LTP) (Takacs *et al.*, 1987 ▶) as a fundamental tool for the inspection of optics. It should be noted that metrology does not only provide characterization of optical components, but such data can be directly used in realistic beamline modelling (Samoylova *et al.*, 2009 ▶).

## 2. On the precision of optical elements to guide and focus X-rays   

Synchrotron optical components are long shaped (up to 1.3 m) and used under the grazing-incidence condition (Wolter, 1952 ▶) which makes them difficult to measure and thus to manufacture. The impact of shape imperfections at optical components in the long spatial frequency error regime (from about 1 mm to aperture length) on the imaging performance of an X-ray focusing system is related to the induced local phase shifts in the reflected beam. The latter distort the wavefront and cause the converging beam to have phase errors. Assuming a small source and a large distance between source and mirror, the acceptable p.v. mirror height variations 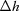 are limited to a few single nanometres only,
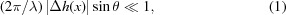
with θ being the grazing angle and λ the wavelength of the X-ray beam (Samoylova *et al.*, 2009 ▶). A further criterion for a beamline performance is the r.m.s. wavefront distortion which should be σ_r.m.s._ < λ/14 or better. This leads to a condition known as the Maréchal criterion (Maréchal, 1947 ▶), where the acceptable root-mean-square height error for a number of optical components over all spatial frequencies present within the residual surface errors is given by
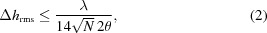
where *N* is the number of reflecting surfaces in the system (Siewert *et al.*, 2012 ▶). Clearly, the requirements on surface quality become linearly more difficult to achieve with decreasing X-ray wavelength, hence the difficult challenge of making hard X-ray reflective optics of sufficient quality. Practically, mirrors of sub-nm r.m.s. figure quality for the long-, mid- and high-spatial figure error need to be manufactured and hence measured. These conditions have been the motivation to improve deterministic finishing technology as well as metrology capabilities. To demonstrate the current state of metrology, we will discuss the inspection of a super-polished focusing mirror pair for beamline P06 at PETRA III. Finishing technology like ion beam figuring (IBF) (Schindler *et al.*, 2003 ▶; Thiess *et al.*, 2010 ▶) and elastic emission machining (EEM) (Yamauchi *et al.*, 2002 ▶) allow the substrate topography to be controlled on an atomic scale. Of course, this is only realistic if precise topography data are available. Mirrors finished by use of EEM have recently shown <1 nm r.m.s. figure accuracy on a length of up to 350 mm (corresponding to 50 nrad r.m.s. slope deviation) (Siewert *et al.*, 2012 ▶) and have allowed diffraction-limited focusing at the CXI experiment at LCLS (Boutet & Williams, 2010 ▶). However, upcoming optics like the 1 m-long KB-focusing mirrors (Kirkpatrick & Baez, 1948 ▶) at the European XFEL with a required residual slope error of 20 nrad r.m.s. (this corresponds to about 1 nm p.v. figure error!) will represent a challenge for both metrology and finishing technology. To reach such a quality, the gravitational sag and clamping forces need to be accounted for in the mirror substrate so as to provide the required figure shape when mounted at the beamline. In addition, diffraction-limited sources translate into very high power densities on optical components and additional care is required to preserve the mirror shape as well as possible, even by means of active optics. Whereas heat-load-induced deformations are very difficult to study *ex situ*, good results have been obtained using at-wavelength metrology (Rutishauser *et al.*, 2013 ▶).

## 3. The principle of slope-measuring deflectometry   

**Figure 2:**
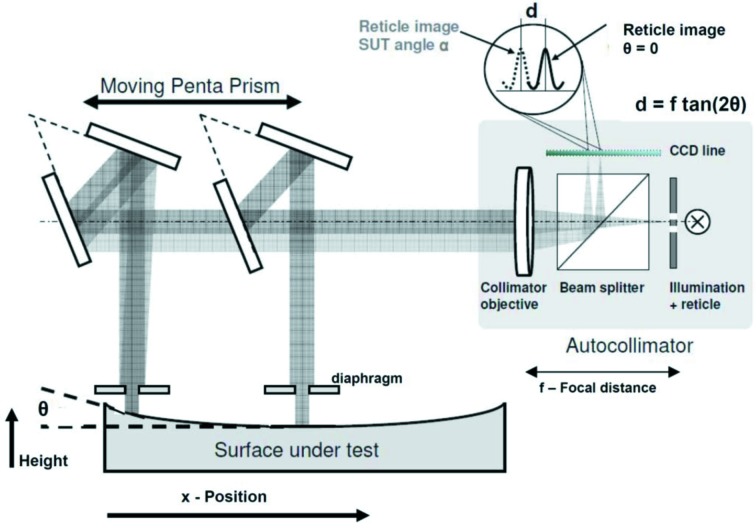
Design of an autocollimator-based slope-measuring profiler.

Slope measuring profilers enable the measurement of flat and slightly curved reflective surfaces by direct measurement of the deflection angle of a probing laser beam. This allows a non-contact measurement non-damaging to the delicate optics. Their advantage over an interferometric system is that they do not rely on external reference surfaces. However, also in the case of slope measuring profilers a careful analysis of the instrument error budget and a calibration of the system are mandatory to achieve the required accuracy (Yashchuk *et al.*, 2007 ▶; Siewert *et al.*, 2010 ▶). In this paper we describe the design of the BESSY-NOM slope-measuring profiler as shown in Fig. 2 ▶. The BESSY-NOM follows the principle of a scanning penta-prism set-up (Qian *et al.*, 1995 ▶), which has shown advantages compared with the classical LTP-II design with a moving detector. To avoid the measurement being influenced by inhomogeneity of a bulk prism, two λ/100 mirrors in a 45° configuration are used to guide the measurement beam onto the surface under test. To achieve an optimal performance the 45° beam-guiding optics are aligned according to a dedicated alignment procedure (Barber *et al.*, 2011*a* ▶,*b* ▶). The NOM is equipped with two optical sensors: a LTP-III head (a design by Peter Takacs) and a modified autocollimator (Siewert *et al.*, 2004 ▶). The NOM autocollimator has been shown to provide a higher accuracy compared with the LTP-III head of the NOM (Siewert *et al.*, 2005 ▶). The laser test beam is traced at regular intervals over the mirror along the line of inspection. Depending on the local topography, the test beam will be reflected into the position-sensitive detector of the NOM autocollimator head. Its position on the CCD line of the sensor is directly related to the local surface slope (see Fig. 2 ▶). The reflection of the test beam along the optical axis of the instrument is determined by the angle 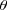 between the mirror normal and the direction of the impinging laser beam (Irick, 1992 ▶; Signorato & Sanchez del Rio, 1997 ▶). Then the local slope *S* is given by
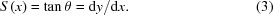
The relative slope change is measured by scanning along the line of inspection. The sensor detects the change of the angle of reflection from one position *x* on the mirror substrate to the next position *x* + Δ*x*. Fig. 2 ▶ shows the optical set-up for the scanning penta-prism configuration by use of an autocollimator as sensor at the NOM. A diaphragm placed at a distance of 3 mm from the optics under test defines the size of the measuring beam. The diaphragm has to be positioned close to the optics under test to avoid the influence of diffraction effects on the measurement. A spatial integration of the slope data finally gives the topography profile *h*(*x*_*k*_),
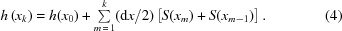



The residual figure error is obtained after the subtraction of an ideal profile, *e.g.* in the case of the focusing mirrors, discussed in this paper, given by an elliptical fit based on the geometrical parameters as defined by the experimental set-up. When using an aperture size of 2.5 mm at the diaphragm, the spatial period range covered by NOM is from 1.7 mm to the full mirror length (Siewert *et al.*, 2013 ▶). Virtually, any curved reflective optical shape can be measured by the NOM as long as the slope change is within an acceptance angle of ±5 mrad. A larger slope change along a line of inspection can be measured by use of stitching techniques as long as the local curvature of the surface under test is larger than about 5 m.

The double CCD array set-up of the autocollimator provides a two-axis angle coordinate system (horizontal and vertical), which allows the measurement of a surface in face-to-the-side or face-up condition by use of the second CCD-array at the same optics head; only a simple change in the beam-guiding mirror-based penta-prism (MBPP) set-up is required. This option is realised at the NOM by a dedicated horizontal MBPP, which must be aligned at the scanning carriage in order to change the scanning beam path from a vertical to a horizontal reflection, thus allowing the measurement of optics under real operating conditions as regards the relative orientation of the optical surface relative to the gravity vector (Siewert *et al.*, 2012 ▶).

## 4. On the limits of slope-measuring deflectometry: alternative options   

In the previous section we have discussed the potential of slope-measuring deflectometry in terms of accuracy. However, like most techniques, slope-measuring deflectometry has some drawbacks. If high spatial resolution beyond the millimetre range is required, interferometric techniques or scanning force microscopy (often named atomic force microscopy, AFM) are the methods of choice. Fizeau interferometers provide a spatial resolution of a few tens of micrometres while white-light interferometers reach about 250–300 nm depending on the objective magnification applied. White-light inteferometry (WLI) combined with stitching techniques (Kimura *et al.*, 2010 ▶) allows the measurement of reflective optics with high spatial resolution up to a length of several 100 mm but suffers from the problem that the stitching period contributes to the error budget; also, possible parabolic errors need to be cross-checked with other measurements. Scanning force microscopy allows the resolution of spatial frequencies down to about 10 nm but this strongly depends on the geometry of the tip. As noted in §3, the radius-of-curvature limit for slope measurements is about 5 m for the autocollimator-based NOM and is about 1 m for the LTP (Siewert *et al.*, 2005 ▶). This forms a limitation for the inspection of, for example, strongly curved mirrors of toroidal or cylindrical shape. These mirrors may have sagittal radii of a few tens of millimetres only. In this case, a measurement by use of an interferometer with a spherical reference allows the inspection of the radius of curvature by measuring the distance between the cats-eye and confocal position. Additionally, the spherical reference allows the measurement of short stripes on such a mirror in the sagittal direction. The achievable accuracy is of course limited by the quality of the reference sphere in use. State-of-the-art spherical references show a quality of λ/50 today (with λ = 633 nm).

## 5. Characterizing optics by use of slope mapping   

**Figure 3:**
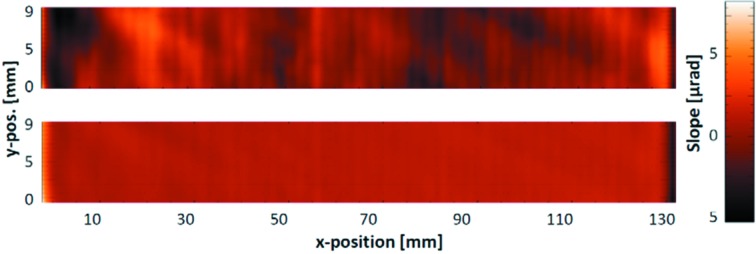
Slope map of an elliptical-cylinder-shaped focusing mirror, before (top) and after (bottom) ion beam figuring. The ellipse parameters are: entrance arm length, 9000 mm; exit arm length, 350 mm; angle of incidence, 2.5°.

A standard slope-measuring profiler allows the measurement of a surface by a single line scan. This is sufficient to provide an acceptance test for synchrotron optics due to the usual long aperture size in the meridional direction and a short aperture width of 1–3 mm in the sagittal direction. Highly accurate three-dimensional topography measurements of an optical surface are required if optical elements are to be characterized in detail or re-worked to optimize them to a more perfect shape. Also, alignment conditions causing a deformation of the optics by careless clamping can be analyzed this way. Initially, we have developed a three-step ‘union jack’-like method to scan the complete surface of a rectangular sample (Siewert *et al.*, 2005 ▶). To generate a three-dimensional-data matrix, two sets of surface scans, each consisting of a multitude of equidistant parallel sampled line scans, have to be traced orthogonally to each other in the meridional and sagittal directions successively. Each single surface line scan is taken on the fly in the forward direction (by one). Between two single line scans the sample is moved laterally by a Y-position carriage. In a final step the two diagonals have to be measured as two individual lines. In this way, a twisting of the surface, which is recognized and measured in the direct measurement, is identified and compensated by data treatment. This method allows a highly accurate mapping of the optics but is rather time-consuming and demands an excellent alignment procedure between the partial measurements. A straightforward and faster option is to measure the optics in the meridional direction only, and to average a set of several scans (6–10) taken at the same line of inspection in a forward–backward mode, which eliminates the spherical error part if the optical element is drifting during the measurement. A forward–backward–backward–forward mode allows the parabolic error contribution to be suppressed (Yashchuk, 2009 ▶). Providing such topography data allows the optics to be improved by, for example, IBF technology. Fig. 3 ▶ shows two results of slope mapping before and after an iteration of ion beam figuring on an elliptical-cylinder-shaped focusing mirror installed at beamline UE49 at BESSY-II, serving a RICXS (resonant inelastic coherent X-ray scattering) experimental station with a 0.6 µm × 4.0 µm focal spot (Könnecke *et al.*, 2013 ▶). The mirror was optimized to a residual slope error of 0.6 µrad r.m.s. compared with 1.65 µrad r.m.s. in its initial state, an improvement by a factor of three.

## 6. Ultra-precise KB mirrors for diffraction-limited focusing at PETRA III   

**Figure 4:**
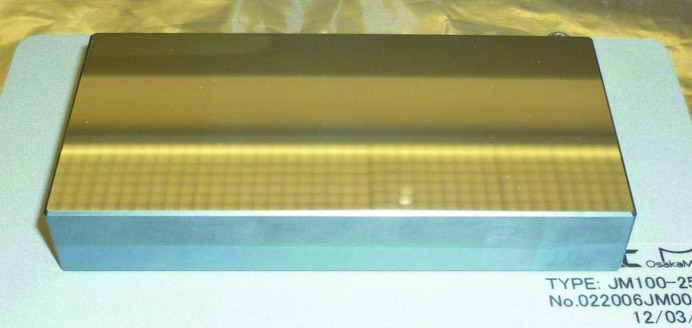
Horizontally focusing mirror at beamline P06 at PETRA III.

To benefit from the excellent properties of a source like PETRA III at DESY, highly precise optical components are required. The Hard X-ray Micro/Nano-Probe beamline P06 at PETRA III uses KB mirrors for focusing in the microprobe experiment and nanofocusing lenses in the nanoprobe experiment (Schroer *et al.*, 2010 ▶). The aim of the microprobe is to provide a flexible experimental set-up with high flux for fast X-ray fluorescence, spectroscopy, diffraction mapping, tomography with sub-micrometre spatial resolution and, in particular, to provide ample space for sample environments and detectors. The KB mirrors are designed to offer a working distance of 200 mm between the edge of the KB mirror and the focus. A high-energy cut-off at 23 keV is provided by a 2.7 mrad grazing-incidence angle of the mirrors and Rh coating. A simple optics scheme without pre-focusing was chosen where the undulator source is demagnified by the KB mirrors and in the basic mode of operation only a cryo-cooled Si111 or multilayer monochromator and a polished diamond exit window is in the beam. The KB mirrors have fixed shape and are super-polished, which is considered to provide the best optical quality and stability. The excellent source sizes of PETRA III (σ_hor_ = 36 µm, σ_vert_ = 6 µm, low-beta section) and the beamline and KB dimensions given below result in a diffraction-limited vertical beam size of 125 nm and a geometrically limited horizontal beam size of 230 nm (FWHM). The ideal shape of such mirrors can be described as an arc of an ellipse. The two foci of the ellipse are the source point of the beamline in the undulator and the focal point at the experimental station. In the KB configuration one mirror focuses in the horizontal direction and the other in the vertical direction. Beamline P06 at PETRA III is equipped with a KB-focusing pair made by EEM technology. These mirrors were inspected at the BESSY-II Optics laboratory before installation. The two silicon substrates are 15 mm thick and of 100 mm in length and 50 mm in width, coated with rhodium. The aperture size is 90 mm × 5 mm. Fig. 4 ▶ shows a photograph of the mirror. Only the central aperture section is coated with Rh. Tables 1 ▶ and 2 ▶ give the mirror parameters as specified and measured.

**Figure 5:**
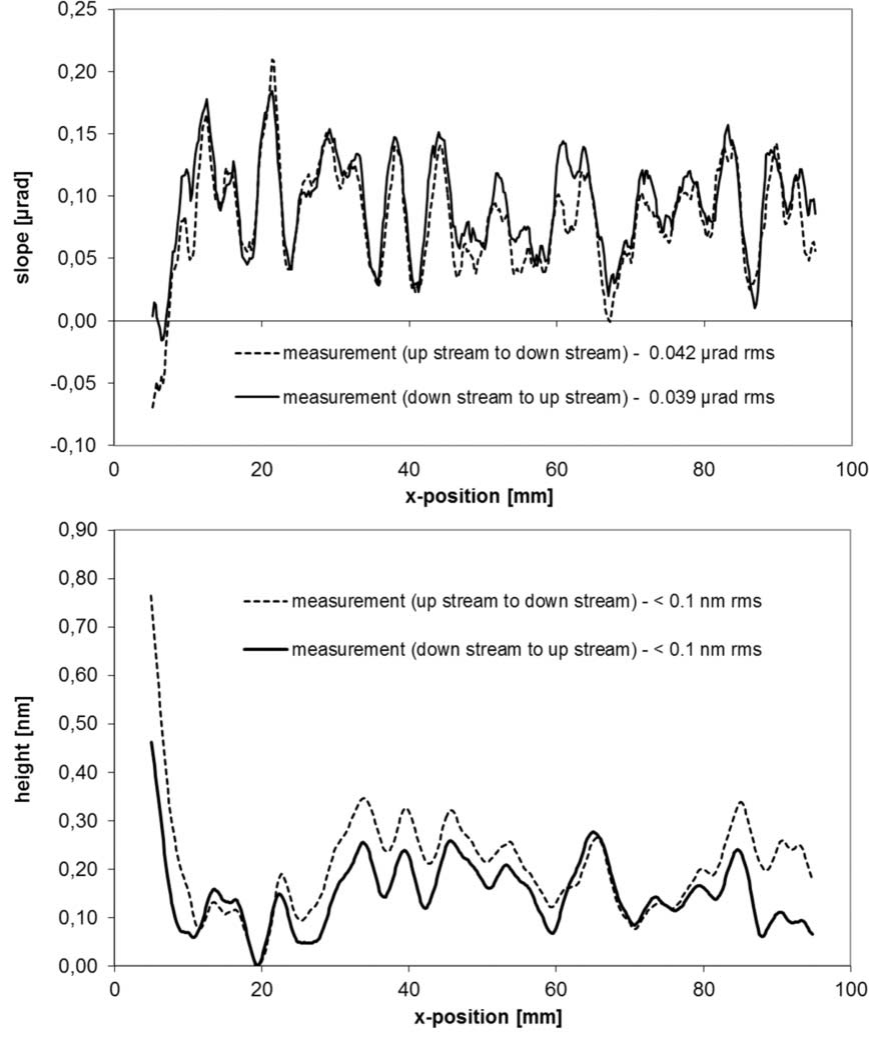
Profile of residual slope (top) and residual height (bottom) for the vertical focusing mirror at beamline P06 at PETRA III.

The micro-roughness of the mirrors was measured with a white-light interferometer (WLI) using a magnification of 1.25 for the mid-spatial-frequency roughness (MSFR) characterization. Magnifications of 20 and 50 cover the high-spatial frequency roughness (HSFR) range. For both the MSFR and HSFR the mirror roughness shows excellent values below 0.2 nm r.m.s. The slope profile of the mirrors was measured by use of the BESSY-NOM in face-up condition along the central line in the meridional direction with a discrete sample spacing of 0.2 mm. To avoid numerical integration errors, a fit has been applied to the measured profile of the slope by varying the ellipse parameter: the entrance and exit arm length as well as the grazing angle until the profile of lowest residual slope deviation is found. An exact equation of the slope of an ellipse is described by Sutter *et al.* (2010 ▶) (see also Sawhney *et al.*, 2010 ▶). For both mirrors a residual slope error of about 40 nrad r.m.s. is measured, showing mirrors of excellent quality (see Figs. 5 ▶ and 6 ▶). Corresponding to this, the profiles of residual height show a result of <0.1 nm r.m.s./0.8 nm p.v. for the vertical focusing mirror (VFM) and <0.1 nm r.m.s./0.37 nm p.v. for the horizontal focusing mirror (HFM). To estimate the error budget for both measurements, the line of inspection was measured first in the upstream–downstream direction and after a 180° horizontal flipping in the downstream–upstream direction. Comparing the results, an agreement of 14 nrad r.m.s. for the HFM and 16 nrad r.m.s. for the VFM was found. This corresponds to an r.m.s. height value of 48 and 56 pm, respectively. These values can be assumed as the estimated level of uncertainty for the measured residual slope and figure error result. It is noted here that this method does not allow the identification of the spherical part in the error budget for the absolute (unfitted) data. The estimated uncertainty for the entrance arm and exit arm length is below 0.1 mm. This is non-critical from a practical point of view and can be easily compensated for by slight variations of the grazing angle during the optics alignment at the beamline. Note that careless clamping of these optical components can cause a significantly higher mirror deformation than the level of uncertainty given by metrology during inspection of the optics (Siewert *et al.*, 2012 ▶).

## 7. On the characterization of multilayer coatings   

**Figure 7:**
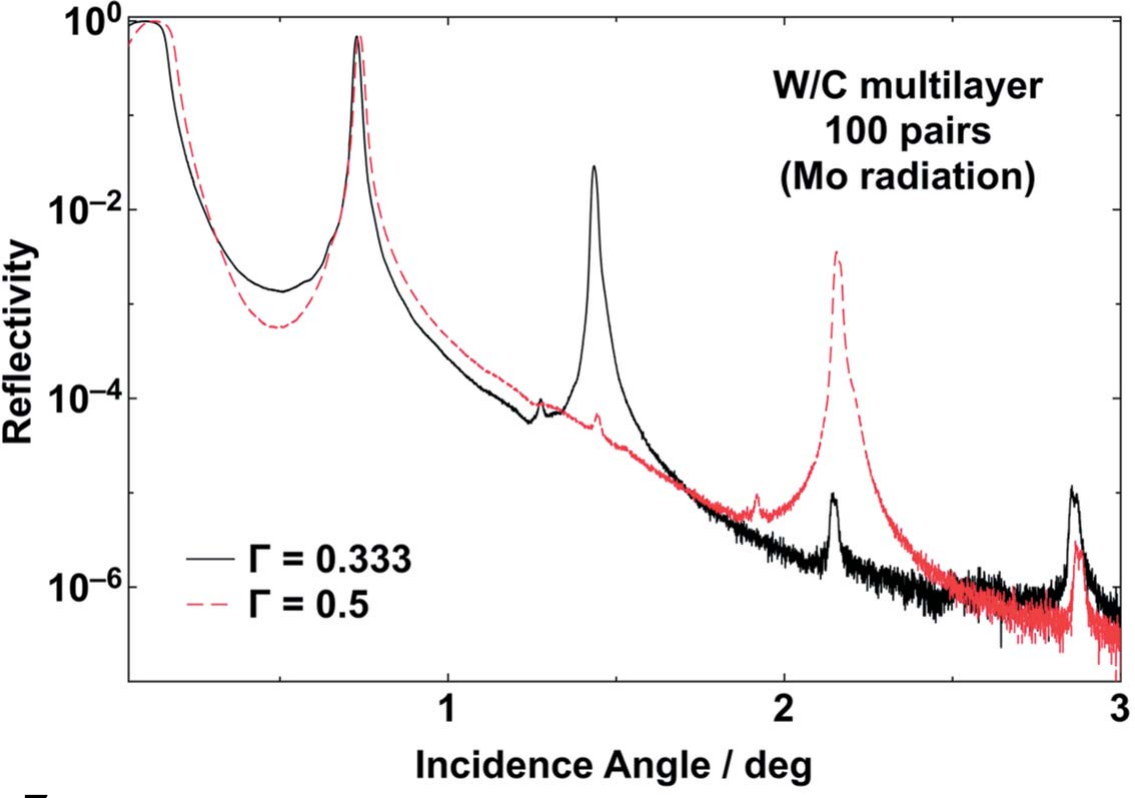
Reflectivity scans of two multilayer mirrors with 100 pairs of W/C, a multilayer period of 2.8 nm and a thickness ratio of 1/3 (solid line) and 1/2 (dashed line), measured with Mo radiation (17.4 keV) at the X-ray reflectometer at Helmholtz Zentrum Geesthacht.

**Figure 8:**
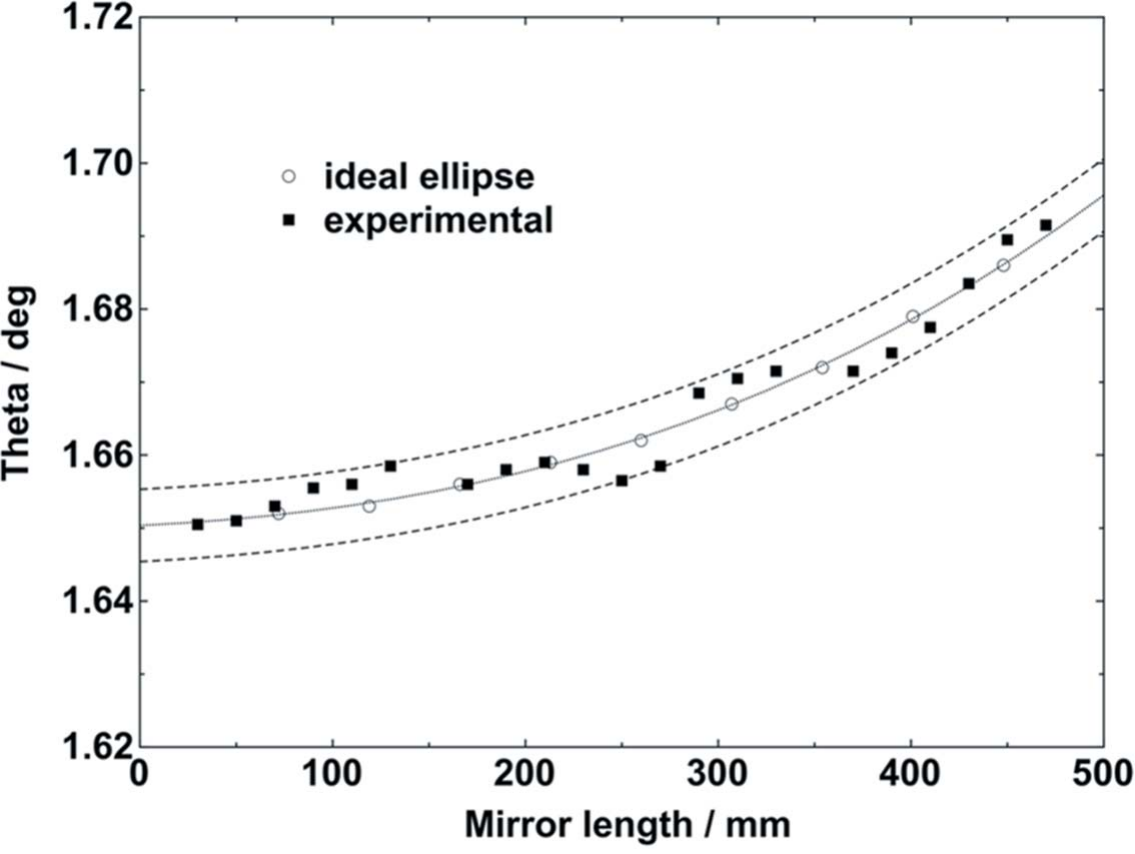
Variation of the Bragg angle along the mirror length of a W/C multilayer with 150 pairs using Cu radiation (8.04 keV) using the X-ray reflectometer at Helmholtz Zentrum Geesthacht; measured multilayer period of 2.75 nm at the left side and 2.68 nm at the right side.

During the last three decades, the demand for multilayer mirrors has increased, first for laboratory sources and later for beamlines at synchrotron storage rings and XFELs (Spiller, 1994 ▶; Attwood, 1999 ▶). Single layers are limited to shallow incidence angles, which are determined by the used photon energy. The reflectivity of single layer coated mirrors is only high below the critical angle. At higher angles, multilayer mirrors provide again high reflectivity due to Bragg reflection. Then, the wavelength range is narrow, which is often desired for X-ray applications such as tomography or diffraction. State-of-the-art magnetron sputtering techniques allow the deposition of highly uniform single layers such as carbon and boron carbide in the sub-nanometre range having a roughness typically below 0.3 nm r.m.s. A p.v. value in layer thickness of less than 2 nm has been achieved on a deposition length of more than 1 m (Störmer *et al.*, 2011 ▶). In the case of multilayers, it is possible to synthesize a stack of layers, which are often called reflector and spacer. The sum of both thicknesses is the period, which determines the Bragg angle in dependence of the used photon energy. The selection of the materials takes into account the optical constants of the possible materials (Henke *et al.*, 1993 ▶). After thin-film deposition, the multilayers are investigated by means of X-ray reflectometry using angular scans (Holy *et al.*, 1999 ▶). In the case shown here, the X-ray characterization was performed using a laboratory diffractometer (D8 Advance; Bruker), which is equipped with a reflectometry stage and a knife edge. A Göbel mirror behind the source was used to shape a parallel X-ray beam. In Fig. 7 ▶, two typical reflectivity scans as a function of the incidence angle are shown, using Mo radiation (17.5 keV). The ratio of the tungsten reflector material thickness to the period is chosen to be 1/2 and 1/3, which is visible in the reduction of the second or third order of the Bragg peaks, respectively. Since the average material density is smaller in the second multilayer, the critical angle is reduced. Using simulations, it is possible to compare measured results and calculated reflectivity and take into consideration important parameters such as thickness, roughness, density of each layer and the number of layers (Windt, 1998 ▶). Fig. 8 ▶ shows the measured Bragg angle of a W/C multilayer along the tangential direction of a 500 mm-long plane X-ray mirror. The X-ray reflectometry measurements were performed using Cu radiation (8048 eV). Across the length of the mirror, the period of the multilayer varies gradually. The challenge was to achieve an elliptical gradient regarding the θ angle. The error band of the θ angle gradient along the mirror length is ±0.3%. The maximum change in thickness is 70 pm over 500 mm, which represents a lateral thickness gradient of about 10^−8^ along the beam direction. It can be stated that current multilayer fabrication on the picometre level reveals a lot of interesting opportunities.

## 8. Outlook and conclusions   

It has been shown that ultra-precise mirrors of 50 nrad r.m.s. residual slope error and better are state-of-the-art today. These values for the residual slope deviation correspond to a height deviation of a few Å r.m.s. on a macroscopic length scale of 90 mm. The characterization of reflective optical components by use of slope-measuring deflectometry enables a detailed diagnostic of the optics topography with spatial resolution from 1.7 mm to aperture length. Available topography data support an improvement of the optics shape if required. However, future optical elements will require comparable and even higher precision down to 20 nrad r.m.s. on significantly larger aperture sizes of up to 1000 mm in length. To enable a characterization of such components with sufficient accuracy, the improvement of the instrument performance as well as the measurement environment will be essential. Thus additional equipment for instrument characterization and calibration is of importance. Future improvement is expected by an European Joint Research cooperation on angle calibration tools (see Acknowledgements).

In multilayer fabrication it is expected that the mirror length will increase further. Simultaneously, demands on mirror performance will be higher for the European XFEL.

Additionally, the physical stability of the complete system, including metrology device, optical components and support mechanics, will become a topic of high relevance. Future laboratory space for metrology will have to be installed in a separate basement, where the influence of noise is reduced. The thermal stability of 20 mK/24 h as realised for the NOM environment at the BESSY-II Optics laboratory is estimated to be sufficient for future work. However, the current environmental conditions at synchrotron radiation beamlines are by far worse than realised at metrology laboratory spaces. This may impact the optics performance at the beamlines and can be only verified by *in situ* monitoring.

Applying advanced measurement strategies will further increase the achievable accuracy in *ex situ* metrology for X-ray optics (Yashchuk, 2009 ▶).

## Figures and Tables

**Figure 6:**
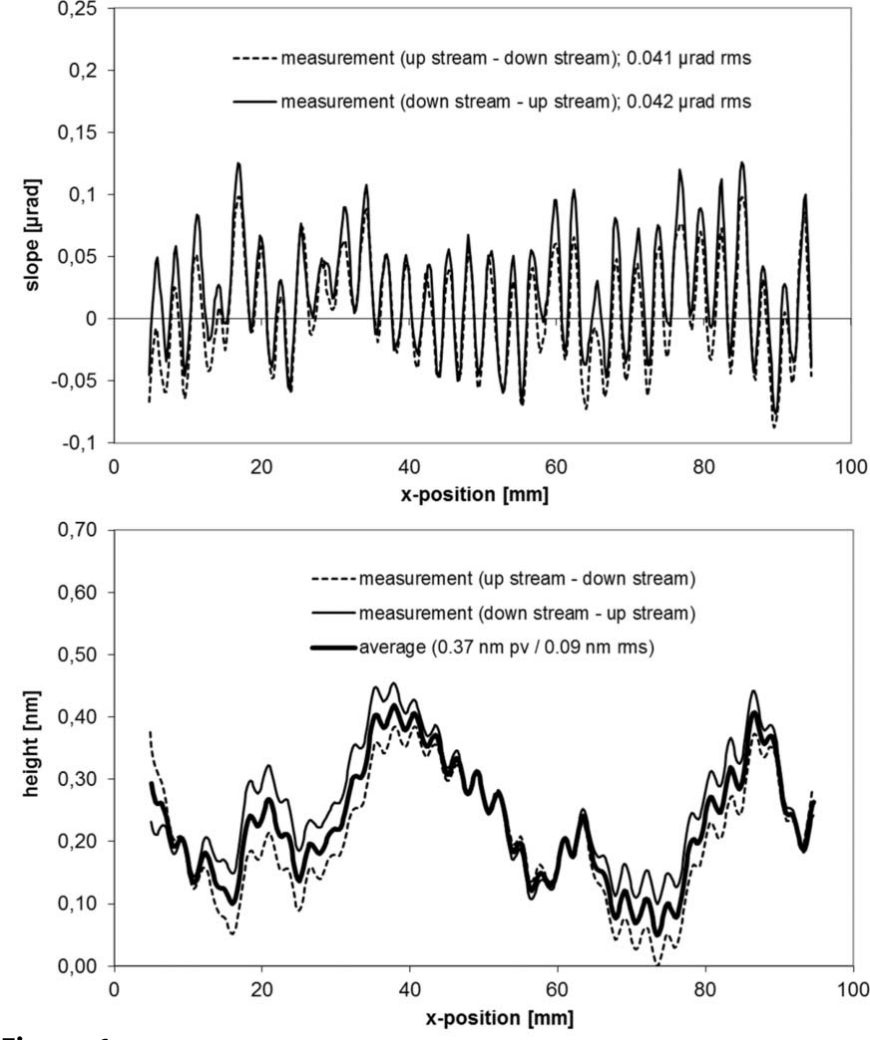
Profile of residual slope top and residual height bottom for the horizontal focusing mirror at beamline P06 at PETRA III.

**Table 1:** Parameters of a vertical KB-focusing mirror VFM at beamline P06 at PETRA III

Mirror parameter	Specification	Measurement result
Source distance (mm)	93595	93595
Focus distance (mm)	355	355
Grazing angle (mrad)	2.7	2.74
Residual slope error (nrad)	Not defined	40
Residual figure error (nm)	p.v.: 1	p.v.: 0.8; r.m.s.: <0.1
Micro-roughness (nm r.m.s.)	*S*_*q*_ ≤ 0.2	*S*_*q*_ = 0.11–0.15 (1.25×)
		*S*_*q*_ = 0.07–0.08 (20×)
		*S*_*q*_ = 0.06–0.109 (50×)

**Table 2:** Parameters of a horizontal KB-focusing mirror HFM at beamline P06 at PETRA III

Mirror parameter	Specification	Measurement result
Source distance (mm)	93700	93700
Focus distance (mm)	250	250
Grazing angle (mrad)	2.7	2.69
Residual slope error (nrad)	Not defined	41
Residual figure error (nm)	p.v.: 1	p.v.: 0.37; r.m.s.: <0.1
Micro-roughness (nm r.m.s.)	*S*_*q*_ ≤ 0.2	*S*_*q*_ = 0.07–0.12 (1.25×)
		*S*_*q*_ = 0.08–0.11 (20×)
		*S*_*q*_ = 0.07–0.10 (50×)
